# Targeting Sensory and Motor Integration for Recovery of Movement After CNS Injury

**DOI:** 10.3389/fnins.2021.791824

**Published:** 2022-01-21

**Authors:** Ahmet S. Asan, James R. McIntosh, Jason B. Carmel

**Affiliations:** Departments of Neurology and Orthopedics, Columbia University, New York, NY, United States

**Keywords:** sensorimotor integration (SMI), spinal cord, motor cortex, movement recovery, paired stimulation, plasticity

## Abstract

The central nervous system (CNS) integrates sensory and motor information to acquire skilled movements, known as sensory-motor integration (SMI). The reciprocal interaction of the sensory and motor systems is a prerequisite for learning and performing skilled movement. Injury to various nodes of the sensorimotor network causes impairment in movement execution and learning. Stimulation methods have been developed to directly recruit the sensorimotor system and modulate neural networks to restore movement after CNS injury. Part 1 reviews the main processes and anatomical interactions responsible for SMI in health. Part 2 details the effects of injury on sites critical for SMI, including the spinal cord, cerebellum, and cerebral cortex. Finally, Part 3 reviews the application of activity-dependent plasticity in ways that specifically target integration of sensory and motor systems. Understanding of each of these components is needed to advance strategies targeting SMI to improve rehabilitation in humans after injury.

## Part 1: Sensorimotor Integration in Health

### Sensorimotor Integration

**Figure 1:**
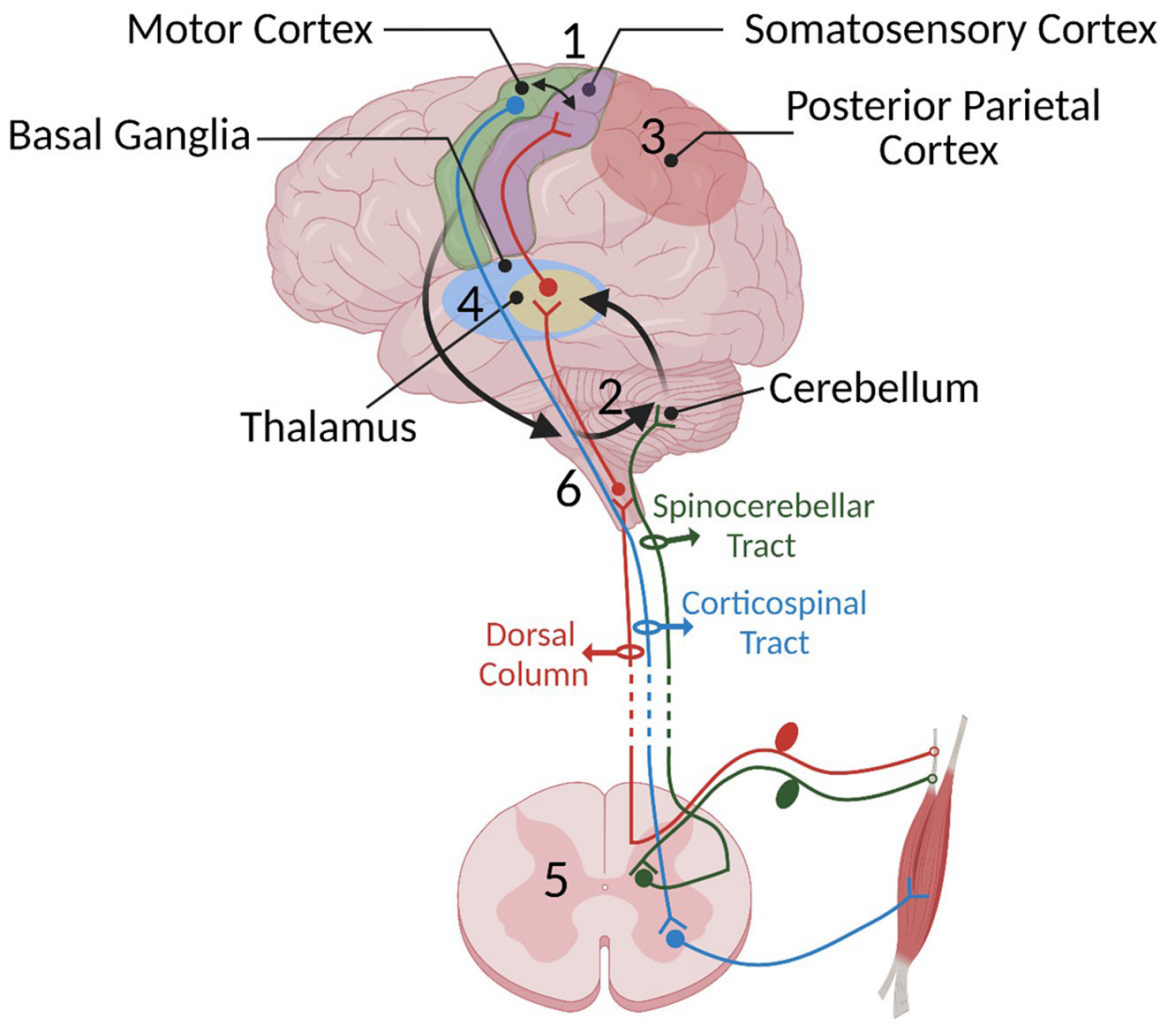
Some of the important SMI centers and their connections mentioned in Part 1: (1) the sensorimotor cortex, (2) cerebellum, (3) posterior parietal cortex, (4) basal ganglia, (5) spinal cord, and (6) brainstem. Sensory information travels to the spinal cord *via* sensory afferents and then is relayed to the sensory centers in the cortex, thalamus, and cerebellum through the dorsal medial lemniscus (shown in red) and spinocerebellar pathway (shown in dark green), respectively. We have chosen not to show the spinothalamic pathway since it has not yet been shown to be crucial to SMI. The cerebellum constantly receives somatosensory information and integrates these senses with an efference copy of motor commands relayed through pontine nuclei located in the brain stem (shown with black arrows) to estimate the sensory consequences of movements. In turn, it provides feedback to the cortical areas through the thalamus. The motor cortex has loops with different cortical areas including basal ganglia, PPC, cerebellum, and brainstem. Information provided by these loops is used to shape the final motor command and the output is sent to the spinal cord. Descending cortical information synapses either directly with lower motor neurons or spinal interneurons. The motor output travels through the ventral root of the spinal cord to muscles to generate movement.

Skilled movement requires coordinated neural activity of the sensory and motor systems. Their timed co-activation is used to converge sensory and motor inputs in the sensorimotor integration (SMI) centers and aims to promote plasticity at their intersection. Sensorimotor integration is the process of incorporating sensation about one's body and the external environment to shape movement (Wolpert et al., 1998a). This process occurs in several areas of the nervous system, including the spinal cord, thalamus, basal ganglia, cerebellum, and several areas of cerebral cortex (Figure 1). Coordinated activity of the sensory and motor systems enables execution of skilled tasks and learning new skills. Injury of these structures impairs movement, in part by disrupting SMI. This paper will review the main tenets of SMI in order to ask whether therapy can exploit these processes to restore function after injury.

**Figure 2:**
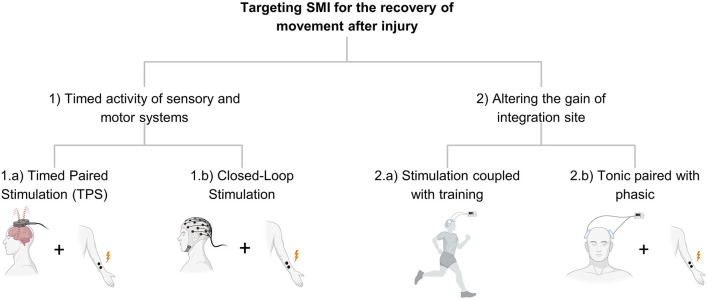
Shows the organization in Part 2 and describes the different strategies of stimulation targeting SMI.

We conceive of therapies directed at SMI as belonging to two main groups. The first is the coordinated activity of sensory and motor systems. Timed engagement of these systems through either endogenous activity (e.g., movement) or exogenous activity (induced by sensory or electrical stimulation) is a consistent theme of therapies targeting SMI. The second type of therapy targets where the two systems meet so that SMI is altered because of altering the gain of the integration site. Rather than altering multiple inputs to a site, these interventions change the state of the single site where integration occurs (Figure 2).

### Relearning of Sensorimotor Functions

Recovery of movement after injury or disease can involve similar processes to motor learning in health. One main difference to relearning after injury is that the circuits available for recovery are limited. A central strategy for targeting SMI after injury is to strengthen the circuits weakened by injury or to use complementary pathways to take over the function of those lost due to injury. This section reviews how we learn movement and the circuits that mediate that learning.

Motor learning is a complex process that requires highly coordinated cascades of processes to acquire and retain skilled movements. Fitts and Posner describe the three stages of motor learning; cognitive, associative, and autonomous (Fitts and Posner, 1967). When one first performs a movement, it is slow with a high error rate, and it requires active cognitive processes. With more practice, associations among cues are developed for the movement and it becomes more fluent and less conscious. At the final phase, movement becomes more accurate and controlled largely without active cognition. In a recent study, these three stages of learning were monitored with fMRI during a sensorimotor adaptation task (Kim et al., 2015; Weaver, 2015). During the first phase, the frontal and parietal cortical regions were most active. In the intermediate phase, anterior regions of the inferior parietal lobe became more involved. Finally, with practice, the cerebellum became highly activated. Even though the fMRI data can only resolve temporal coactivation on the scale of seconds, it provides the locus of the coactivation of sensory and motor systems. Here, we will focus on the role of the sensorimotor cortex, posterior parietal cortex (PPC), cerebellum, basal ganglia, spinal cord, and finally brainstem-motor pathways as complementary regions that are involved in this sensorimotor process (Figure 1).

The central nervous system (CNS) also uses different computational methods to promote learning: supervised, unsupervised, and reinforcement learning (Doya, 1999). Different brain regions such as the cerebellum, basal ganglia, and cerebral cortex are specialized to use certain kinds of computation and learning (Doya, 1999, 2000; Raymond and Medina, 2018). We will also discuss some of these mechanisms in more detail under relevant sections.

#### 1) Sensorimotor Integration in the Cortex

The cortical contribution to SMI is tuned by its connections to subcortical structures such as the thalamus and to areas within the cortex. After receiving peripheral somatosensory inputs, the thalamus relays that information primarily to layer 4 (L4) of the somatosensory cortex (S1) and also L4 of the primary motor cortex (M1) (Yamawaki et al., 2014; Barbas and García-Cabezas, 2015). The neurons in this layer have an excitatory projection within the hemisphere to L2-3 in the M1 and S1 (Yamawaki et al., 2014). Information flows between primary sensory and motor cortices largely through horizontal connections in L2–3. The axonal connection from sensory cortex onto neurons within motor cortex in this layer is the site for plasticity and sensorimotor integration (Kaneko et al., 1994a,b) and also essential for acquiring skilled movement (Papale and Hooks, 2018).

Sensory feedback enables skilled sensorimotor behavior, but the relatively long time needed for the feedback to reach sensorimotor centers can make adjustments slow (Miall and Wolpert, 1996). Use of an efferent phenocopy likely speeds the adjustment of the movement from forward model centers and is necessary for some skilled movement (Azim et al., 2014). The same motor command used to perform a movement is also delivered to the sensorimotor system such as the PPC and cerebellum, and the sensory feedback is interpreted in relationship to this plan (Wolpert et al., 2011). This model suggests that the CNS predicts the sensory consequences of motor movement and uses it to decrease the movement error, known as feedforward control. In this context, practice becomes crucial since it optimizes the internal mechanisms when the prediction and the sensory feedback do not overlap (error detection). That also means that sensory feedback updates the forward model in order to modify the motor plan, and this assists in generating a faster and more accurate movement. Learning in the CNS also improves motor performance and reduces the need for correction. Information processed in the cerebellum and cortex seems to play an essential role in this model.

#### 2) Cerebellum

The cerebellum is critical for feedforward control (Wolpert et al., 1998b). It is thought to receive the efferent copy of the motor command through the cortico-ponto-cerebellar pathway and somatosensory information through the spinocerebellar tract. It also receives vestibular (Ango and Dos Reis, 2019) and visual information (Glickstein, 2000). This sensory and motor integration in the cerebellum is important for various sensorimotor tasks including coordination and postural control. Incoming sensory and motor information updates the cerebellum about the motor plan and the current state of the body. So, a prediction for the sensory consequence of the movement is made and feedback to cortical regions involved in the motor plan is made through the cerebello-thalamic-cortico pathway. It also receives the actual sensory inputs to check the prediction error. If an incorrect prediction was made by the cerebellum, the synaptic connections also need to be modified. In this regard, it is thought that the inferior olive, located in the medulla, provides the error signal to the cerebellum and modulates the synaptic connections of the Purkinje cells (supervised learning) (Ishikawa et al., 2016; Medina, 2019). Purkinje cells generate the sole output from the cerebellar cortex and project onto the deep cerebellar nuclei. Then, the deep cerebellar nuclei send the cerebellar output to cortical regions involved in motor plan and execution.

The specific sites for SMI in the cerebellum have been partially described. Proville et al. provided direct evidence of sensorimotor integration in the cerebellum at the cellular level by showing the convergence of the cortical sensory and motor inputs in the cerebellar crus 1 region during whisking behavior in rats (Proville et al., 2014). Likewise, the cerebellum also modifies the sensorimotor integration in the cortex. Popa et al. showed that cerebellar inactivation disrupts the gamma coherence between the sensory and motor cortex (Popa et al., 2013a) which has been shown to be critical for attention, memory, and associative learning (Miltner et al., 1999; Jensen et al., 2007).

Cerebellar contribution to SMI is not limited to its connection with the cortex. Vestibulocerebellar and spinocerebellar loops are also critical for SMI. For example, SMI is important for the vestibulo-ocular reflex (VOR) which allows stabilizing gaze during head movement to ensure stable vision (Cullen, 2012). This reflex adapts to changing sensory information. However, cerebellar damage removes the adaptability of VOR to these changes (Li et al., 1995; Ito, 1998). This result also supports the role of the cerebellum for error reduction during motor learning. The spinocerebellar tract carries somatosensory information to the cerebellum, and impairment in this pathway also dramatically affects SMI and impairs skilled movement, balance, and posture (MacKinnon, 2018).

#### 3) Posterior Parietal Cortex

The PPC plays a pivotal role in creating an internal model of the outside world (Blakemore and Sirigu, 2003; McNamee and Wolpert, 2019). It receives and integrates the different sensory modalities from the somatosensory, visual, and auditory systems to generate the representation of the current state of the body and its environment. It contributes to several sensorimotor functions such as motor planning, visually guided locomotion, and eye movement (Konen and Kastner, 2008; Marigold and Drew, 2011). Previously, the direct connection of the PPC with M1 was in question. However, studies on non-human primates showed the presence of reciprocal connections between PPC and M1 and the premotor cortex (Fang et al., 2005). These findings are also supported by recent human studies in which paired PPC and M1 stimulation was applied and optimum latency was measured for altering the cortically evoked MEPs. The strongest facilitation was observed at 2–4 ms (Koch et al., 2007, 2009; Karabanov et al., 2012). The short ISI indicates a monosynaptic connection between PPC and M1.

Posterior parietal cortex is heavily involved during the early phases of sensorimotor behavior. In the Karabanov et al. study, participants performed a sensorimotor task while cortical activity was recorded with EEG. They showed that interaction between M1 and PPC increased during the early phases of training and decreased in the late phases (Karabanov et al., 2012). Similar to the cerebellum, it is thought that the PPC receives the efference copy of the motor command and integrates this with the sensory information, and provides feedback to the motor cortex (Blakemore and Sirigu, 2003; Koch et al., 2008; Cui, 2016). The main differences between the cerebellum and PPC are the phase of motor learning during which they participate and the role in execution. Posterior parietal cortex appears to be more engaged in the early stages of sensorimotor learning and is also thought to be more involved in movement planning and goal selection (Mulliken et al., 2008; Aflalo et al., 2015). The cerebellum, on the other hand, participates more in late phases, and it contributes to the rapid prediction of the sensory consequences of movement (Blakemore and Sirigu, 2003; Hull, 2020).

#### 4) Basal Ganglia

The basal ganglia processes sensory and motor inputs from the cortex and shapes motor output. It receives somatotopically organized inputs from the motor cortex, premotor cortex, supplementary motor area, primary somatosensory cortex, and superior parietal lobule (Lanciego et al., 2012). After processing information from these multiple sources, it modulates the activity of the thalamus that in turn projects to the cortex. It does not receive direct sensory information from the periphery. The basal ganglia processes the signals through two different pathways: direct and indirect. At rest, it has an inhibitory tone in the thalamus (Fischer, 2021). The direct pathway removes the inhibition on the thalamus, and increases the motor cortex activity. The output of the indirect pathway, on the other hand, suppresses the thalamus to prevent unwanted movements. The balance of activity between the direct and indirect pathways is modulated by dopamine (Gerfen and Surmeier, 2011). Cells of the direct pathway predominantly have excitatory D1 and the indirect pathway has inhibitory D2 receptors (Gerfen et al., 1990; Sian et al., 1999). Hence, dopamine suppresses activity in the direct pathway and facilitates the indirect pathway.

The basal ganglia are critical for action selection (Gurney et al., 2001a,b; Friend and Kravitz, 2014). They have different parallel competing loops with several different cortical areas such as the motor cortex, prefrontal cortex, and limbic areas (Leblois et al., 2006; Leisman et al., 2014; Aoki et al., 2019). These different loops provide the necessary content for movement and help the basal ganglia to shape cortical output (Gurney et al., 2001a; Leisman et al., 2014). For example, the loop with the limbic system is implicated in an emotional component, and the motor cortex provides information about the motor plan. It is thought that the basal ganglia select the appropriate loop for a specific movement by increasing its activity while decreasing the activity of other loops (Gurney et al., 2001a,b; Prescott et al., 2006).

Introducing a reward is critical for this selection, which also suggests a critical role of basal ganglia in reward-based learning (Doya, 1999; Doya et al., 2001). Schultz et al. showed in primates that dopamine neurons initially respond to the reward after successful trials (Schultz et al., 1993; Schultz, 1998). Once animals learned the task, dopamine neurons stopped responding to the reward; instead, they responded to the conditioned visual stimulus applied before the delivery of the reward. Thus, dopamine activity is not just important for the present reward but also prediction of the future reward, a critical component of reinforcement learning (Tanaka et al., 2016). This learning mechanism in the basal ganglia allows action selection by assessing capacity for reward.

#### 5) Spinal Cord

The spinal cord is the termination of motor output and the initial entry point for somatosensation, and it serves as a critical node of SMI. The spinal cord receives input from pyramidal neurons in layer 5 located in the premotor cortex, primary motor cortex, and primary and secondary somatosensory cortices directly *via* corticospinal tract (CST), and indirectly through brain stem projections. These descending motor pathways project onto the alpha motor neurons either directly or indirectly through premotor interneurons in the intermediate zone of the spinal cord. In humans, inputs from the somatosensory cortex project extensively onto the interneurons located in dorsal and lateral regions of the spinal cord while primary motor cortex inputs project more ventrally.

Interneurons integrate descending motor commands and segmental somatosensory information. As an example of how the spinal cord can accomplish complex movement, spinalized animals without any brain to spinal cord connections can walk and even recover from a stumble, all with spinal circuits alone (central pattern generators) (Whelan, 1996; Côté et al., 2018). This mechanism allows the spinal cord to contribute to generating rhythmic movement patterns such as walking and swimming (Marder and Bucher, 2001). Central pattern generators do not require sensory information for generating movement; however, sensory feedback is necessary to fine-tune motor output. In addition to coordinating movement, interneurons modulate reflexes and somatosensation. The incoming sensory afferents are presynaptically inhibited by interneurons whose activity is also controlled by descending cortical inputs (Flanders, 2011). This mechanism is named primary afferent polarization (PAD). This procedure occurs in the cord in a very selective manner to filter the sensory inputs (Eguibar et al., 1994, 1997) and is shown to be a crucial mechanism for voluntary movement (Hultborn et al., 1987; Seki et al., 2009).

Spinal cord circuitry also plays a vital role in controlling respiratory muscles (Monteau and Hilaire, 1991; Gad et al., 2020) and is involved in haemodynamic stability (Squair et al., 2021). Further understanding of this circuitry is also important in developing stimulation where targeting SMI might also play a significant role in modulating these activities.

#### 6) Brainstem-Motor Pathways

Descending motor control is exerted both directly *via* the corticospinal system as well as indirectly through cortex to brainstem to spinal cord connections. As one of the important descending pathways located in the brainstem, the rubrospinal tract originates within the midbrain and contributes to SMI at the spinal level (Moreno-López et al., 2016). It arises from the red nucleus and receives inputs from different brain areas including cortex, *via* cortico-rubral tract, and the cerebellum (Wyart and Knafo, 2015). Rubrospinal inputs converge with CST in the spinal segmental level at interneurons and propriospinal neurons which are also receiving cutaneous and muscle afferents (Olivares-Moreno et al., 2021). The rubrospinal tract works in parallel with CST and is important for skilled movement. It plays a critical role in various SMI functions including posture (Zelenin et al., 2010), gait (Lavoie and Drew, 2002), and control of skilled movements of forelimb digits (Küchler et al., 2002). Its enhanced input to the cord also compensates for the loss of movement after CST lesion (Ishida et al., 2019).

The reticulospinal tract also originates in the brainstem and contributes to SMI in the spinal cord. It receives inputs from different sources containing somatic, vestibular, and cerebellar information. Reticular projection to the spinal cord is shown to reduce voluntary reaction time (Rothwell, 2006). In humans, voluntary reaction time can be modified by a startle cue (Brown et al., 1991; Valls-Solé et al., 1995). Patients with CST injury show greater startle response also indicating enhanced reticulospinal influence on the spinal cord to compensate for the loss of CST function (Ballermann and Fouad, 2006; Sangari and Perez, 2019). Strengthening the reticulospinal connection also improves hand motor function (Zaaimi et al., 2012). Baker et al. used an auditory startle cue and combined it with gross hand function (including a power grip) after SCI (Baker and Perez, 2017). They reported that auditory cues applied during movement promote the reticular inputs entering the spinal cord and enhance hand function both in healthy participants and SCI patients.

As our understanding of the various brain stem motor systems evolves, they might be targeted for SMI according to their main motor output. For example, the tectospinal pathway for neck movement or the corticorubral pathway for arm and hand control.

### Summary

Sensory-motor integration enables skilled movement using an internal model of the body and its relationship to the environment to plan a movement. That motor plan is used to predict the sensory consequences, and updated with sensory feedback. Sensory-motor integration is a process that is disseminated across centers classically considered motor centers, such as the motor cortex and the ventral spinal cord, and sensory centers, such as the sensory cortex and sensory thalamus. Sensory-motor integration also involves several feedback loops involving the basal ganglia, cerebellum, and others. Knowledge of the nervous system circuits that enable skilled movement through SMI can be used to predict the consequence of injury (Part 2) and also guides application of interventions to strengthen SMI and improve function (Part 3).

## Part 2: Disruption of Sensorimotor Integration by Injury or Disease

Lesion studies have taught us about the roles of the cortex in SMI. As one good example of this, Wolpert et al. studied a patient with a lesion in the superior parietal lobe (Wolpert et al., 1998a). This patient showed impairment in detecting constant tactile and proprioceptive stimuli without visual information (tactile fading), even though she had intact proprioceptive and tactile systems. To investigate the motor consequences of tactile fading, the patient was asked to maintain a precision grip. Without visual feedback, she failed to generate a constant grip force which declined to near zero for about 15 s. The results of this study shed light on the role of PPC in SMI and demonstrated new evidence of its importance in storing inner representations of the body's state. In this part, we will briefly go over studies that assess how an injury or lesion in the sensorimotor centers, in particular the sensorimotor cortex, spinal cord, and cerebellum, affect sensorimotor behavior.

### Injury in the Sensorimotor Cortex

Lesions in either primary or sensory cortices impair motor learning. Animal studies on cats (Sakamoto et al., 1989), monkeys (Pavlides et al., 1993; Liu and Rouiller, 1999), and rats (Kawai et al., 2015) showed that lesion in either one of these regions prevents the acquisition of skilled behavior and impairs sensorimotor learning. However, learned motor movements are not significantly affected by the injury of either the motor or somatosensory cortex (Pavlides et al., 1993; Kawai et al., 2015). These findings agree with others by showing the involvement of subcortical regions in motor learning and motor control (Jueptner et al., 1997; Errante and Fogassi, 2020). The basal ganglia (Foerde and Shohamy, 2011) and cerebellum (De Zeeuw and Ten Brinke, 2015) in particular are likely the locus of movement memory.

Middle cerebral artery stroke damages sensory and motor cortices and, therefore, results in movement impairment (Walcott et al., 2014; Bolognini et al., 2016; Edwards et al., 2019b). Most stroke patients show difficulty with tactile sensation, proprioception, and stereognosis (Winward et al., 1999; Connell et al., 2008). Considering the necessity of sensory information for performing a successful sensorimotor task, deficits in the sensory system directly cause sensorimotor dysfunctions such as disruption in postural control (Dietz, 2002), impairment in temporal (Gentilucci et al., 1994), and spatial (Gordon et al., 1995) movement aspects. Sensory deficits are also observed once the motor cortex is injured. Focal ischemic lesions in monkeys' M1 showed sensory deficits suggesting the disruption in sensorimotor integration (Nudo et al., 2000). Parallel to the lesion studies in animals, stroke patients also show difficulty acquiring new motor skills, and the level of injury is also directly correlated with the degree of impairment in skill acquisition (Cirstea et al., 2003; Boyd et al., 2007).

### Spinal Cord Injury

Spinal cord injury (SCI) impairs movement through the interruption of white matter tracts and the destruction of gray matter at the site of injury. Complete injury means there is no motor or sensory function below the injury site. Patients with incomplete (partial) injury, on the other hand, have some preserved movement or sensation below the injury site. Fortunately, most SCI patients have spared white matter connections in the cord, even in those without preserved function (Kakulas and Kaelan, 2015; Wagner et al., 2018).

Spinal cord injury both damages the intrinsic circuitry in the spinal cord and the necessary sensory and motor information for this circuitry to function properly. Following injury, supraspinal control of the spinal network is largely attenuated. Sensory inputs, therefore, have an outsized influence on the spinal cord below the injury (Caron et al., 2020). As a result, afferent sensory fibers become more active. This in turn leads to disinhibition of spinal reflexes and hyperreflexia. Increased activity of sensory afferents also causes the hyperexcitability of motoneurons (Mailis and Ashby, 1990; Grey et al., 2008). Lack of dexterity, increased muscle tone, and involuntary muscle contractions are also common after SCI (Adams and Hicks, 2005; Nielsen et al., 2007; Holtz et al., 2017). Attenuation of the spinal inhibitory mechanism, hyperexcitability of motoneurons, and lack of supraspinal input in the cord are some of the main reasons causing these dysfunctions.

### Cerebellar Degeneration

Cerebellar degeneration occurs due to the deterioration of the cerebellar neurons, and this increases progressively in many of the common cerebellar diseases. This degeneration could result from either genetic (Paulson, 2009) or non-genetic causes (Sullivan et al., 1995). Lesions in other CNS areas such as spinal cord can also impair the cerebellar circuitry due to loss of critical inputs (Visavadiya and Springer, 2016; Lei and Perez, 2021). As a result, cerebellar patients manifest uncoordinated movement along with deficits in motor adaptation/learning such as visuomotor learning and adaptations in walking and reaching (Schlerf et al., 2013; Martino et al., 2014).

Diseases affecting cerebellar processing also alter its influence on other CNS regions. Ming Kuei Lu et al. showed that repetitive paired cerebellum and motor cortex stimulation causes lasting changes in motor cortex excitability (Lu et al., 2012). However, this modulatory effect dissipated in patients with Parkinson's disease and spinocerebellar ataxia (Lu et al., 2017). Cerebellar-cortex inhibition was also abrogated in those patients. Similarly, others showed that activity in the cerebellum modulates the plasticity in the brain induced by paired motor cortex-peripheral nerve stimulation (Hamada et al., 2012; Popa et al., 2013b). This facilitatory effect is disrupted in patients with cerebellar degeneration (Dubbioso et al., 2015). As another approach, repetitive stimulation of peripheral nerves also adjusts motor cortex excitability (Kaelin-Lang et al., 2002; Luft et al., 2002). It has been postulated that sensory inputs directly act in the motor cortex to generate this effect. However, this observed effect disappears in rats with removal of the controlling half of the cerebellum (Nordeyn et al., 2005; Taib et al., 2005). This highlights that cerebellar processing of sensory information is essential for this form of cortical plasticity (Luft et al., 2005).

### Summary

Central nervous system injury and disease perturbs sensorimotor integration through disruption of nodes of the network or by their disconnection. The type of movement disturbance ranges from loss of fine control to paralysis, depending on the location and severity. The pattern of injury also helps to determine the substrate to target for therapy (Part 3). In many cases, this will be the node of the network that was disrupted. However, other therapies target intact circuits that are intended to take over the functions of the injured ones.

## Part 3: Targeting SMI for the Recovery of Movement After Injury

In the Introduction, we described two main approaches to targeting SMI for recovery of movement: timed activity of sensory and motor systems, and strengthening the sites where integration occurs (Figure 2). In this section, we describe interventions that target SMI, either explicitly or implicitly. In these descriptions, we try to identify the biological processes involved. For interventions involving electrical stimulation, we describe either phasic stimulation, a short period of stimulation that is timed to activity in another system, or tonic stimulation, which is meant to alter the excitability over a period of time, usually minutes. Phasic stimulation is typically involved in processes requiring tightly coordinated or timed stimulation of the two systems. In contrast, tonic stimulation targets the excitability of one system to enable stronger interaction with the other.

### Timed Activity of Sensory and Motor Systems

#### Timed Paired Stimulation

Timed paired stimulation (TPS) involves stimulation at two different sites so that they arrive synchronously at one of the sites for SMI (Figure 2, 1.a). These types of interventions arise from an understanding of the basic properties of neural systems involved in learning. Spike timing-dependent plasticity (STDP) is one mechanism that the sensorimotor system utilizes to adjust the connection between pre- and post-synaptic neurons contingent on their relative firing pattern. With STDP, repeated firing of a presynaptic neuron a few milliseconds prior to a postsynaptic neuron enhances the synaptic connection between them, known as long-term potentiation (LTP) (Caporale and Dan, 2008). If the postsynaptic neuron fires first, then the synapse is weakened, known as long-term depression (LTD).

The first clinical application of TPS was paired associative stimulation (PAS) (Stefan et al., 2000; Carson and Kennedy, 2013). This stimulation paradigm involves repeated pairing of motor cortex and median nerve stimulation; for each pair of stimuli, the peripheral electrical stimulation is delivered milliseconds before motor cortex activation with transcranial magnetic stimulation. The direction of the observed modulatory effect, LTP or LTD-like plasticity, depends on the interstimulus interval between the cortical (motor) and peripheral (sensory) stimulation. ISIs of 20–25 ms (PAS25) lead to lasting facilitation of cortical excitability whereas 10 ms (PAS10) generates a suppressive effect (Classen et al., 2004). Sensory evoked potentials to median nerve stimulation are observed in somatosensory cortex about 20 ms after stimulation (Allison et al., 1991). Considering the additional time necessary for the S1–M1 interaction, this result suggested that motor cortex is the site of interaction for the facilitation effect (Suppa et al., 2017).

Whereas spike-timing dependent plasticity relies on the relative activity of an input neuron and a receiving neuron, plasticity can also be induced by the coordinated activity of multiple inputs onto a common target (Harel and Carmel, 2016). This mechanism seems to be widely employed by the sensorimotor system to induce plasticity. For example, the cerebellum plays a critical role in eyeblink conditioning, one of the most studied associative learning paradigms. In this paradigm, an unconditioned stimulus, causing a motor response, is paired with a neutral sensory stimulus called a conditioned stimulus. After repetitive pairing of these two stimuli with a certain time delay (Wetmore et al., 2014), the motor response is generated as a response to the conditioned stimulus alone. The sensory responses to conditioned and unconditioned stimuli arrive in the cerebellum from pontine nuclei and inferior olive, respectively (Cheng et al., 2015). It is postulated that these inputs converge onto two different targets, Purkinje cells and the deep cerebellar nuclei, and induce plasticity at both sites (Freeman and Steinmetz, 2011).

Similarly, spinal afferents and descending cortical tracts have largely overlapping terminals onto spinal interneurons. We tested whether electrical stimulation of these two inputs could alter the excitability of the cervical spinal cord. We repeatedly paired subthreshold spinal cord stimulation (Capogrosso et al., 2013; Sharpe and Jackson, 2014; Greiner et al., 2021), targeting sensory afferents, and suprathreshold motor cortex stimulation at a latency that caused them to converge in the spinal cord. This paradigm, called spinal cord associative plasticity, strongly increased spinal cord excitability (Mishra et al., 2017). Inactivation of either of the spinal inputs blocked the augmentation of spinal excitability and showed the necessity of these two pathways for plasticity (Pal et al., 2020). Thus, TPS can alter SMI with either STDP or convergent mechanisms.

#### TPS for Stroke

Stroke patients manifest aberrations in their sensory and motor connections which impairs sensorimotor integration (Bolognini et al., 2016; Edwards et al., 2019b). Much of the brain stimulation work to date has focused on balancing interactions between the injured (stroke) hemisphere and the uninjured hemisphere (Hummel and Cohen, 2006; Webster et al., 2006). Following stroke, excitability decreases in the injured hemisphere, while the inhibitory connections from the uninjured hemisphere remain. Thus, the uninjured hemisphere can be seen to “bully” the injured hemisphere, and this imbalance correlates with degree of movement impairment (Carmel and Friel, 2016). Stimulation methods used for stroke mostly act on the motor cortex to restore balance by increasing the excitability of the injured cortex or reducing interhemispheric inhibition by suppressing the activity of the uninjured hemisphere. In this regard, one-site stimulation such as rTMS (Hao et al., 2013), and tDCS (Elsner et al., 2016) have used these mechanisms to promote partial recovery after stroke.

Since stroke is a sensorimotor disorder, paired stimulation of sensory and motor systems can also be a good candidate to directly target sensorimotor integration to restore movement. After the publication of PAS in healthy volunteers, paired peripheral nerve and motor cortex stimulation was used in therapeutic trials. As one of the first studies that employed paired stimulation for stroke, Uy et al. applied 30 min paired suprathreshold motor cortex stimulation with a train of peroneal nerve stimulation repeatedly every weekday for 4 weeks. Participants included in this study received physical therapy, but they did not show any functional improvement for at least 6 months prior to PAS. Pairing strengthened the evoked potentials from cortex and improved gait, including cadence, stride length, and time to heel strike scores (Uy et al., 2003). These results also showed the potential of PAS in the treatment of chronic cases.

Cortical stimulation is also paired with muscle stimulation for movement recovery. Castel-Lacana et al. applied paired cortical TMS and a pulse train electrical stimulation to extensor carpi radialis muscle for 30 min in people with subcortical stroke (Castel-Lacanal et al., 2009). Cortical stimulation was applied 25 ms after the end of each train 1, 5, 12 months after injury. The strongest increase in MEPs occurred after 5 months, and there was still an increase in MEPs after 12 months. The Fugl-Meyer score for wrist and finger extension and the strength of wrist extension showed significant improvement at 5 and 12 months. These results suggest a time after stroke when PAS is effective.

The results of these studies are supported by recent findings where a relatively larger number of participants were used (Palmer et al., 2018; Silverstein et al., 2019). These studies used paired cortical TMS with peripheral nerve stimulation (PAS25). Paired associative stimulation increased both the cortical MEPs and motor performance, measured with the serial reaction task and Fugl-Meyer scale. In addition to the studies focusing on directly stimulating the ipsilesional site, Jayaram et al. stimulated the contralesional cortex with inhibitory PAS to balance the abnormal interhemispheric inhibition due to stroke. They reported that suppressing the contralateral cortical activity promoted ipsilesional cortical excitability and increased MEP response recorded from paretic limb (Jayaram and Stinear, 2008).

#### TPS for SCI

Spinal cord injury damages the connection between the periphery and the brain and causes severe motor and sensory dysfunction. Fortunately, most people with SCI have spared connections in the cord (Kakulas and Kaelan, 2015; Wagner et al., 2018), and these sparse connections can be recruited. For example, stimulation of the intact CST after injury to the other side improved skilled movement and triggered sprouting of spinal axons in rats (Brus-Ramer et al., 2007; Carmel et al., 2010; Carmel and Martin, 2014). This suggests that even sparse innervation can be used to restore function if it is properly engaged.

The intact spinal circuitry below the injury is a target for recovery of movement (Ling et al., 2020; Zavvarian et al., 2020). Timed paired stimulation studies for SCI pair cortical stimulation either with peripheral or spinal cord stimulation. Bunday et al. combined the motor cortex and antidromic stimulation of the ulnar nerve for 17 min and targeted the spinal cord as a site for STDP (Bunday and Perez, 2012). They showed that corticospinal transmission, index finger force, and EMG amplitude increased for up to 80 min. Index finger abduction during the intervention also further improved this observed effect (Bunday et al., 2018). In a subsequent study, the same stimulation paradigm was used but this time peripheral stimulation targeted the peroneal nerve for lower limb function. Once the antidromic volleys evoked by peripheral stimulation reached the spinal cord after cortically evoked presynaptic volleys, it caused an increase in MEPs for 30 min in SCI patients (Urbin et al., 2017). They proposed that observed effects resulted from the strengthening of the corticospinal-motoneuronal synapses. This effect could also have resulted from the recruitment of the antidromic (motor) pathway or the orthodromic sensory pathway together with descending motor pathways.

The Ahmed lab has also demonstrated that peripheral stimulation can be combined with motor cortex stimulation to alter spinal excitability and restore function after SCI (Ahmed, 2010; Ahmed and Wieraszko, 2013). A critical lesson from these studies is that the pairing can target convergence at several levels, and targeting several together can increase the size of modulation.

In our laboratory, we explicitly targeted SMI in the spinal cord by pairing motor cortex stimulation with dorsal spinal cord stimulation. In rats, pairing suprathreshold cortical and subthreshold spinal stimulation generates a significant increase in MEPs, but only if they are timed to converge in the spinal cord (Mishra et al., 2017). When the properly timed stimulation is repeated over 5 min, there is lasting augmentation of cortical and spinal MEPs, which we call spinal cord associative plasticity. Stimulation of the motor cortex or spinal cord alone, or paired stimulation at an inappropriate latency did not alter excitability. Importantly, inactivation of either the CST or the large-diameter afferents from adjacent spinal levels fully abrogated the paired stimulation effect, demonstrating the necessity of these connections (Pal et al., 2020).

Spinal cord associative plasticity was effective to improve the physiology and function in rats with contusion SCI. Rats were injured at the C4 level, and 10 days later 30 min repetitive pairing was applied for the subsequent 10 days. For physiology, a lasting increase in the excitability of both motor cortex and spinal cord was observed, but hyperreflexia was reduced. Rats with paired stimulation significantly outperformed rats with only injury on a test of forelimb dexterity (Pal et al., 2020). Thus, targeting SMI in the spinal cord produced adaptive changes in rats with cervical SCI.

#### Closed-Loop Stimulation

Another important question that needs to be resolved for the timed paired stimulation paradigm is whether the endogenous activity (Figure 2, 1.b) of the brain or externally evoked brain activity (Figure 2, 1.a) provides more effective stimulation for sensorimotor repair. Functional electrical stimulation (FES), which applies electrical muscle stimulation to generate contraction in paralyzed muscles, is used to strengthen muscle responses and restore motor behavior after injuries such as SCI and stroke. Recently, chronically implanted cortical electrodes were used to decode brain activity for movement intent, and this information was used to control muscle stimulation both in animals (Moritz et al., 2008; Ethier et al., 2012) and humans (Bouton et al., 2016; Ajiboye et al., 2017) with SCI. These studies reported significant progress in restoring hand and limb functions. However, one disadvantage of repetitive muscle stimulation is that it can lead to uncoordinated movements and/or muscle fatigue (Thrasher et al., 2005; Jackson and Zimmermann, 2012; Zimmermann and Jackson, 2014).

Directly targeting the neuronal pools in the spinal cord can prevent these unwanted effects and potentially provide more coordinated movements. On this basis, Nishimura et al. controlled intraspinal stimulation in paretic monkeys using high gamma activity recorded from the motor cortex (Nishimura et al., 2013a). Spinal stimulation was triggered once the local field potentials surpassed a certain threshold. They showed that pairing improved volitional control of upper limb movement. In a consecutive study, the same group also used the spike activity of cortico-motorneuronal cells to stimulate the spinal cord. The results demonstrated a significant increase in MEPs, and STDP mechanism being considered as the likely driver for this effect (Nishimura et al., 2013b). Similarly, Zimmermann et al. used a monkey model and reversibly inactivated the cortical region controlling hand movement (Zimmermann and Jackson, 2014). They obtained neural activity from the premotor cortex for the hand function and decoded it to determine the parameters required to drive spinal cord stimulation, and this resulted in improving hand function. Epidural stimulation has been used to drive activation of spinal afferents that can be timed to converge with descending motor activation. This strategy helped to restore walking after SCI (Capogrosso et al., 2016). Adjusting the spinal cord stimulation parameters based on the cortical activity further enhanced recovery (Bonizzato et al., 2018).

The closed-loop approach is also used to strengthen the connection between sensory and motor regions in the brain. Guggenmos et al. disrupted movement with a lesion in the motor cortex in rats which caused impairment in reaching and grasping ability (Guggenmos et al., 2013). The activity in the premotor cortex was used to stimulate the somatosensory cortex. Two weeks of stimulation markedly increased reaching and grasping functions and returned them to their pre-injury level.

These studies support that stimulation controlled by endogenous activity could be a promising approach to restore sensorimotor functions. This method can also provide high specificity since it adds stimulation to the endogenous activation of the neural circuits that normally enable a specific movement. Direct comparison of this technique with TPS could also provide a further understanding of which methods are more effective to potentiate plasticity for recovery after injury.

#### Operant Conditioning of Spinal Reflexes

The spinal cord has also been targeted for SMI through coactivation of descending motor and segmental reflexes. Wolpaw et al. have unraveled the brain centers that can condition the H-reflex, making it larger or smaller with operant conditioning (Wolpaw, 1987; Chen and Wolpaw, 1995; Carp et al., 2006). Many different brain regions contribute to the descending brain influence on the long-term modulation of reflexes, including motor cortex (Chen et al., 2006a; Balakrishnan and Ward, 2013), basal ganglia (Takakusaki, 2017), and cerebellum (Chen and Wolpaw, 2005; Wolpaw and Chen, 2006; Matsugi et al., 2014). This approach has been used to improve function in rats (Chen et al., 2006b, 2014) and humans (Thompson et al., 2013; Thompson and Wolpaw, 2021) with SCI. In contrast to volitional modulation of segmental reflexes, the Edwards lab used transcranial magnetic stimulation to modulate soleus reflexes (Cortes et al., 2011). This also promoted lasting changes in spinal excitability. These studies demonstrate that descending motor pathway stimulation, whether endogenous or exogenous, modulates spinal cord, including spinal cord reflex circuitry in a lasting way. In addition, this modulation is distributed in several supra spinal centers.

#### TPS for the Cerebellum

The level of activity in the cerebellum alters the excitability in the sensorimotor centers such as the cortex and spinal cord. In order to modify the effects of PAS, cerebellum stimulation has been used to alter the gain of cortical plasticity. Stimulation was applied to the cerebellum to adjust its activity level and combined with PAS (Hamada et al., 2012; Popa et al., 2013b). The modulatory effect of cerebellum stimulation was observed with one PAS protocol (PAS25) but not another (PAS21). The small difference in timing that alters the effectiveness of the cerebellum suggests that PAS may induce plasticity at different sites, and cerebellar stimulation is effective at only one of these. These cerebellar dependent and independent pathways were also demonstrated to be engaged in distinct motor learning processes (Hamada et al., 2014).

The above-mentioned studies only employed healthy participants while others have reported exciting results by showing that cerebellar modulation ameliorates cortical activity after brain injuries such as stroke (Abbasi et al., 2021). Instead of TPS, paired tonic cerebellar and spinal cord stimulation was used in people with ataxia (Benussi et al., 2018). Application of direct current stimulation to both cerebellum and lumbar spinal cord increased all performance scores including finger dexterity and gait speed as well as motor cortex excitability. It also facilitated cerebellar brain inhibition, indicating an increase in the cerebello-thalamo-cortical connections. Previous studies also reported recovery in motor performance with cerebellar tDCS in ataxia (Benussi et al., 2017, 2020); however; a direct comparison of pairing, only-spinal and only-cerebellar stimulation is missing in the literature to investigate the synergistic effects. Since tonic stimulation was employed in this study, it does not inform us regarding the convergence site.

### Strengthening the Sites Where Integration Occurs

#### Stimulation Coupled With Training

Training leads to the formation of plasticity that arises from the intrinsic activity of neural networks as opposed to the externally imposed plasticity *via* stimulation (Green and Bavelier, 2008). Training after injury increases activation of the existing sensorimotor circuitry and causes structural modifications associated with plasticity (Ganguly and Poo, 2013). Both training and stimulation methods share common mechanisms to form plasticity such as enhancing synaptic efficacy using NMDA receptor and cortical excitability (Constantine-Paton, 1990). Thus, combining these two approaches has the potential to generate a synergistic effect and offer more effective treatment for recovery (Figure 2, 2.a).

The effect of training paired with motor cortex stimulation has been studied both in animals and humans. Adkins-Muir et al. used a sensorimotor cortex lesion model in rats (Adkins-Muir and Jones, 2003). They implanted a subdural stimulating electrode over the adjacent motor cortex and applied 50 Hz electrical stimulation concurrently with a forelimb reaching task. Rats that received stimulation with training showed a stronger improvement in forelimb performance, and these animals also expressed larger dendritic growth compared to the training only group. In humans, Brown et al. also applied epidural stimulation to the impaired hand region of the motor cortex and combined it with motor rehabilitation therapy to improve hand and arm function (Brown et al., 2006). Patients who received stimulation with therapy showed significantly better motor performance compared to the therapy-only group. Combining the cortical electric stimulation with paretic hand training also demonstrated an expansion of the hand representation area in the cortex along with a significant behavioral improvement in monkeys (Plautz et al., 2003) and rats (Adkins et al., 2008).

Paired stimulation and training has been used more recently with non-invasive cortex stimulation, such as transcranial direct current stimulation (tDCS). Transcranial direct current stimulation offers ease of use and portability, and it can be applied with training. The primary mechanism of tDCS to modulate neural activity is thought to be to shift the activation of a population of neurons closer to their firing threshold (Giordano et al., 2017). Once it is combined with training, it allows the neural tissue below the stimulating electrodes to become more easily activated. Allman et al. applied anodal tDCS to motor cortex on the side of a stroke during motor training for 9 consecutive days (Allman et al., 2016). They reported enhancements in movement, including dexterity, coordination, muscle strength, and these effects lasted for at least 3 months after the intervention. Functional magnetic resonance imaging (fMRI) results demonstrated marked facilitation in brain activity during movement of the affected hand compared to the control group. Enlargement in gray matter volume is also observed in the stimulation group, and no change was reported in the control group. The functional and anatomical results of this approach are supported by many other groups (Edwards et al., 2009; Lefebvre et al., 2012; Giacobbe et al., 2013; Rocha et al., 2016); however, studies with negative results also exist in the literature (Geroin et al., 2011; Leon et al., 2017; Edwards et al., 2019a). The conflicting results observed in these studies potentially stem from the difference between the experimental groups such as injury severity or age of participants (Straudi et al., 2016).

Timed paired stimulation and training coactivate the sensory and motor systems. To investigate if combining these two modalities generates a synergistic effect, Jo et al. applied paired motor cortex and peripheral nerve stimulation with exercise in people with chronic incomplete SCI (Jo and Perez, 2020). They had three groups; only stimulation, stimulation with exercise, and sham stimulation with exercise. Each group improved performance by decreasing the time used to complete motor tasks. Stimulation with exercise and only stimulation groups were able to increase the motor evoked responses and maximum voluntary contraction in target muscles. Also, behavioral and physiological effects were preserved for at least 6 months only in the stimulation + exercise group. This study suggests that adding training on top of TPS may strengthen the therapeutic effect.

Tonic stimulation of the spinal cord also improve function of people with SCI when combined with exercise. Harkema et al. and Angeli et al. showed in consecutive studies that epidural subthreshold stimulation combined with training help complete SCI patients regain the voluntary control of paralyzed muscles (Harkema et al., 2011; Angeli et al., 2014). They proposed that spinal stimulation modulates the interneurons and motoneurons and enhances excitability of the motor pool in the spinal cord (driving them closer to the threshold) (Gill et al., 2018; Wagner et al., 2018).

Multiple nodes of the sensorimotor network can be targeted to improve SMI. Picelli et al. performed a series of experiments to investigate how cerebral and spinal DCS modulates the recovery obtained with robot-assisted gait training after chronic stroke. In their first study (Picelli et al., 2015), they targeted the ipsilesional cortex and spinal cord as stimulation sites and paired this simulation with gait training. They reported that paired anodal cortical and cathodal spinal tDCS with gait training generated significant increases in the walking distance test compared to either site alone. In a follow-up study, they compared the effect of cerebellar to cerebral stimulation (Picelli et al., 2018). They showed that cerebellar stimulation generated a markedly stronger effect than cortical stimulation when paired with spinal DCS and training. To conclude, multiple site stimulation may offer advantages to improve function over one site alone.

#### Tonic Paired With Phasic

Stimulation protocols coupling tonic and phasic stimulation mostly use tDCS applied over one of the sensorimotor centers such as the cortex or spinal cord and pair it with phasic stimulation targeting either the sensory or motor system (Figure 2, 2.b). The tonic stimulation is aimed to increase responsiveness of the sensorimotor centers to phasic stimulation. These approaches gain circuit selectivity at the intersection of the two stimuli. However, they do so without precise timing, likely by altering the gain of the node being modulated with tonic stimulation.

Celnik et al. paired peripheral nerve stimulation with anodal tDCS on the motor cortex to evaluate its effect in a motor sequence task after chronic stroke (Celnik et al., 2009). The patients received varying combinations of tDCS and PNS in different sessions. Pairing demonstrated better recovery than either intervention alone. In another study, Sattler et al. applied anodal tDCS to the ipsilesional motor cortex and paired it with PNS in unilateral hemispheric ischemic stroke patients (Sattler et al., 2015). Participants were divided into two groups: anodal tDCS+PNS and sham tDCS+PNS. Each group received 20 min of stimulation for 5 consecutive days. Pairing generated a stronger enhancement compared to the sham tDCS+PNS group.

The circuitry in the spinal cord has also been targeted with tonic stimulation and combined with phasic cortical stimulation. Song et al. paired intermittent theta-burst stimulation, targeting the motor cortex, with cathodal spinal tDCS (tsDCS) in pyramidal tract lesioned rats (Song et al., 2016). Animals received stimulation for 27 min a day for 10 days, which started 1 day after the injury. The paired stimulation group showed better performance for walking in the horizontal ladder task along with an increase in cortical excitability. The anatomical analysis also revealed that this paired protocol caused axonal sprouting in the spinal cord, and this was five times larger than that of rats with sham tsDCS.

The same stimulation paradigm improved function after SCI in rats (Zareen et al., 2017). Paired intermittent theta burst stimulation (iTBS) and tsDCS was applied for 30 min daily for 10 days compared to an injury only group. Rats with stimulation showed significantly better performance in the food manipulation task. Stimulation also caused axonal outgrowth both below and above the injury level. This study was replicated by our group (Yang et al., 2019). Our results corroborated the previous findings and showed the effectiveness of this stimulation paradigm to restore skilled walking as well. The results suggest that combined stimulation strengthens spinal cord connections, and this improves skilled movement.

### Summary

Therapy directed at SMI has largely targeted the most accessible nodes of the network, including cortex, cerebellum, and spinal cord. Some interventions have been modeled on mechanisms of learning that require coactivation of the pre- and postsynaptic neuron or two inputs to the same target. Others elevate the receptiveness of a node of the network to learning through tonic stimulation. Finally, combining training with stimulation may enhance sensorimotor learning through synergistic effects. Often the stimulation is delivered to the site of disease or injury, but activation of alternative circuits has also been effective.

## Future Directions

There remain open questions that need to be addressed to develop more effective stimulation methods.

What is the source of variation between studies? Even though encouraging results have been reported by many groups, discrepancies exist in the literature regarding the effectiveness of some stimulation paradigms. The reasons for these differences could result from variation between stimulation groups such as the age of participants, size and site of the lesion, and time interval after injury as well as the specifics of the intervention. Determining the reasons for these differences will define the crucial ingredients for effective therapy. Having blinded studies with large sample size is necessary to determine effect size and how widely applicable an intervention targeting SMI might be.

Should the activity used to modify SMI be endogenous activity or externally evoked neural activity? Both exogenous stimulation alone and closed-loop stimulation methods offer promise to induce plasticity and restore function. Closed-loop systems require more complex engineering, however they could offer more specific modulation since the circuits necessary for movement are the ones activated. On the other hand, specificity in TPS is achieved by timing stimuli to converge on specific sites, and this approach can create a large effect on system physiology. A direct comparison of the two could provide an understanding of the type of activity necessary to adaptively modulate the network.

Should therapy be directed at specific circuits, or should activation of a larger network be performed? The appeal of circuit-specific repair is the possibility of better on-target effects and fewer off-target ones. On the other hand, circuit specificity might be achieved after an intervention with task-specific training. More general approaches may offer stronger effects because of their ability to recruit many circuits simultaneously. Rigorous studies comparing these approaches are needed to determine the functional efficacy.

What is the best site to target for improvement of SMI? The ease of access to the cortex, its central role in acquisition of skilled movement, and accessible techniques to stimulate it have made it the most popular target. Although there is strong evidence that cortical stimulation is effective, effect sizes are often variable across interventions. The cerebellum is another attractive target because it modulates the activity in the other sensorimotor centers and also directly integrates sensory and motor information. Finally, spinal cord modulation has produced impressive gains in function for SCI, with emerging applications to other disease states. It is not known whether the large effects of partial reversal of paralysis are due to the target, per se, or whether the disease state and severe impairment are also important. Again, direct comparison of different sites of stimulation for different disease states is necessary to find the right site of stimulation for each disease state or potentially, even each patient.

Published stimulation modalities targeting SMI have not demonstrated adverse effects. However, there is always the possibility that these interventions could worsen, rather than improve, SMI or pathologies such as spasticity or pain. While the evidence to date is promising in providing reward with little risk, the field should remain vigilant for possible maladaptive effects.

## Author Contributions

All authors listed have made a substantial direct and intellectual contribution to the work. ASA drafted the manuscript and the figures. ASA, JRM, and JBC edited and approved the final version.

## Funding

Research reported in this publication was supported by the National Institute of Neurological Disorders and Stroke of the National Institutes of Health under award number R01NS115470.

## Conflict of Interest

The authors declare that the research was conducted in the absence of any commercial or financial relationships that could be construed as a potential conflict of interest.

## Publisher's Note

All claims expressed in this article are solely those of the authors and do not necessarily represent those of their affiliated organizations, or those of the publisher, the editors and the reviewers. Any product that may be evaluated in this article, or claim that may be made by its manufacturer, is not guaranteed or endorsed by the publisher.
